# Immunoglobulin and T Cell Receptor Genes: IMGT^®^ and the Birth and Rise of Immunoinformatics

**DOI:** 10.3389/fimmu.2014.00022

**Published:** 2014-02-05

**Authors:** Marie-Paule Lefranc

**Affiliations:** ^1^The International ImMunoGenetics Information System^®^ (IMGT^®^), Laboratoire d’ImmunoGénétique Moléculaire (LIGM), Institut de Génétique Humaine, UPR CNRS, Université Montpellier 2, Montpellier, France

**Keywords:** IMGT, immunogenetics, immunoinformatics, IMGT-ONTOLOGY, IMGT Collier de Perles, immunoglobulin, T cell receptor, major histocompatibility

## Abstract

IMGT^®^, the international ImMunoGeneTics information system^®^^1^, (CNRS and Université Montpellier 2) is the global reference in immunogenetics and immunoinformatics. By its creation in 1989, IMGT^®^ marked the advent of immunoinformatics, which emerged at the interface between immunogenetics and bioinformatics. IMGT^®^ is specialized in the immunoglobulins (IG) or antibodies, T cell receptors (TR), major histocompatibility (MH), and proteins of the IgSF and MhSF superfamilies. IMGT^®^ has been built on the IMGT-ONTOLOGY axioms and concepts, which bridged the gap between genes, sequences, and three-dimensional (3D) structures. The concepts include the IMGT^®^ standardized keywords (concepts of identification), IMGT^®^ standardized labels (concepts of description), IMGT^®^ standardized nomenclature (concepts of classification), IMGT unique numbering, and IMGT Colliers de Perles (concepts of numerotation). IMGT^®^ comprises seven databases, 15,000 pages of web resources, and 17 tools, and provides a high-quality and integrated system for the analysis of the genomic and expressed IG and TR repertoire of the adaptive immune responses. Tools and databases are used in basic, veterinary, and medical research, in clinical applications (mutation analysis in leukemia and lymphoma) and in antibody engineering and humanization. They include, for example IMGT/V-QUEST and IMGT/JunctionAnalysis for nucleotide sequence analysis and their high-throughput version IMGT/HighV-QUEST for next-generation sequencing (500,000 sequences per batch), IMGT/DomainGapAlign for amino acid sequence analysis of IG and TR variable and constant domains and of MH groove domains, IMGT/3Dstructure-DB for 3D structures, contact analysis and paratope/epitope interactions of IG/antigen and TR/peptide-MH complexes and IMGT/mAb-DB interface for therapeutic antibodies and fusion proteins for immune applications (FPIA).

## IMGT^®^: The Birth of Immunoinformatics

IMGT^®^, the international ImMunoGeneTics information sys- tem^®^^1^ (1), was created in 1989 by Marie-Paule Lefranc at Montpellier, France (CNRS and Université Montpellier 2). The founding of IMGT^®^ marked the advent of immunoinformatics, a new science, which emerged at the interface between immunogenetics and bioinformatics. For the first time, immunoglobulin (IG) or antibody and T cell receptor (TR) variable (V), diversity (D), joining (J), and constant (C) genes were officially recognized as “genes” as well as the conventional genes (2–5). This major breakthrough allowed genes and data of the complex and highly diversified adaptive immune responses to be managed in genomic databases and tools.

**Figure 1:**
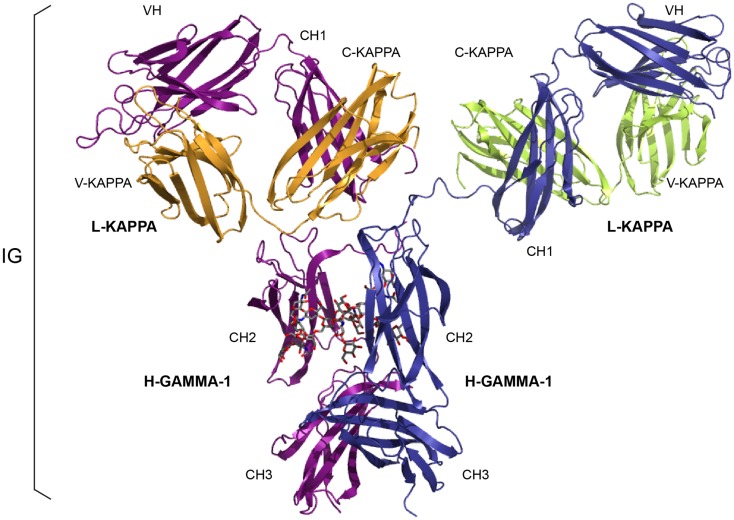
**An immunoglobulin (IG) or antibody**. *In vivo*, an IG or antibody is anchored in the membrane of a B cell as part of a signaling B cell receptor (BcR = membrane IG+CD79) or, as shown here, is secreted (2). An IG is made of two identical heavy (H, for IG-HEAVY) chains and two identical light (L, for IG-LIGHT) chains (2). An IG comprises 12 domains (for example, IgG1, shown here) or 14 domains (IgM or IgE). The V-DOMAIN of each chain and the C-DOMAIN, one for each L chain and three for each H chain are highlighted. The light chain (here, L-KAPPA) is made of a variable domain (V-DOMAIN, here, V-KAPPA) at the N-terminal end and a constant domain (C-DOMAIN, here, C-KAPPA) at the C-terminal end. The heavy chain (here, H-GAMMA-1) is made of a VH (at the N-terminal end) and of three CH (four for H-MU or H-EPSILON) (Table 1) (2). The structure is that of the antibody b12, an IgG1-kappa, and so far the only complete human IG crystallized [1hzh from IMGT/3Dstructure-DB (http://www.imgt.org)].

**Figure 2:**
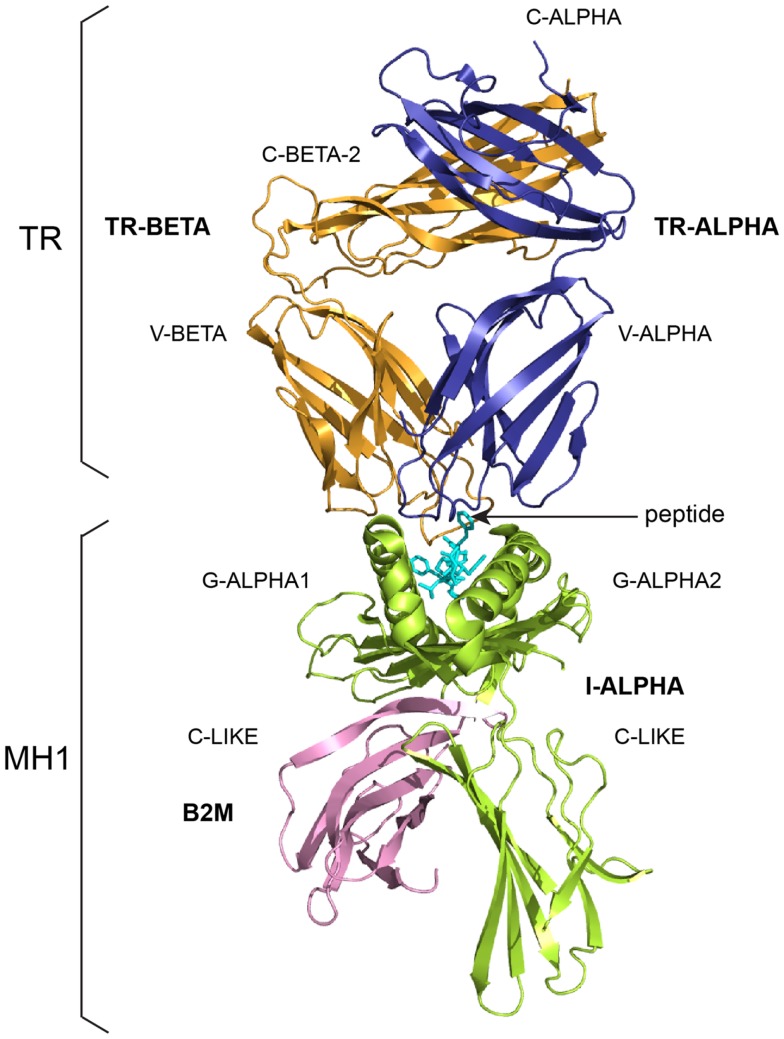
**A T cell receptor (TR)/peptide-major histocompatibility 1 (pMH1) complex**. A TR (here, TR-alpha_beta) is shown (on top, upside down) in complex with an MH (here, MH1) presenting a peptide in its groove. *In vivo*, a TR is anchored in the membrane of a T cell as part of the signaling T cell receptor (TcR = TR+CD3). A TR is made of two chains, each comprising a variable domain (V-DOMAIN) at the N-terminal end and a constant domain (C-DOMAIN) at the C-terminal end (3). The domains are V-ALPHA and C-ALPHA for the TR-ALPHA chain, V-BETA and C-BETA for the TR-BETA chain (Table 2) (3). An MH1 is made of the I-ALPHA chain with two G-DOMAIN (G-ALPHA1 and G-ALPHA2) and a C-LIKE-DOMAIN (C-LIKE), non-covalently associated with the B2M (a C-LIKE-DOMAIN) (8). The TR/pMH1 complex structure is 3qfj from IMGT/3Dstructure-DB (http://www.imgt.org).

The adaptive immune response was acquired by jawed vertebrates (or *gnathostomata*) more than 450 million years ago and is found in all extant jawed vertebrate species from fishes to humans. Understanding the basis for adaptive immunity, at the level of cell populations, individual cells, and molecules, has been a major focus of immunology in the past century (6, 7). The adaptive immune response is characterized by a remarkable immune specificity and memory, which are the properties of the B and T cells owing to an extreme diversity of their antigen receptors. The specific antigen receptors comprise the immunoglobulins (IG) or antibodies of the B cells and plasmocytes (2) (Figure 1), and the T cell receptors (TR) (3) (Figure 2). The IG recognize antigens in their native (unprocessed) form, whereas the TR recognize processed antigens, which are presented as peptides by the highly polymorphic major histocompatibility (MH, in humans HLA for human leukocyte antigens) proteins (Figure 2).

The potential antigen receptor repertoire of each individual is estimated to comprise about 2 × 10^12^ different IG and TR, and the limiting factor is only the number of B and T cells that an organism is genetically programed to produce (2, 3). This huge diversity results from the complex molecular synthesis of the IG and TR chains and more particularly of their variable domains (V-DOMAIN) which, at their N-terminal end, recognize and bind the antigens (2, 3). The IG and TR synthesis includes several unique mechanisms that occur at the DNA level: combinatorial rearrangements of the V, D, and J genes that code the V-DOMAIN [the V–(D)–J being spliced to the C gene that encodes the C-REGION in the transcript], exonuclease trimming at the ends of the V, D, and J genes and random addition of nucleotides by the terminal deoxynucleotidyl transferase (TdT) that creates the junctional N-diversity regions, and later during B cell differentiation, for the IG, somatic hypermutations and class or subclass switch (2, 3).

IMGT^®^ manages the diversity and complexity of the IG and TR and the polymorphism of the MH of humans and other vertebrates. IMGT^®^ is also specialized in the other proteins of the immunoglobulin superfamily (IgSF) and MH superfamily (MhSF) and related proteins of the immune system (RPI) of vertebrates and invertebrates (1). IMGT^®^ provides a common access to standardized data from genome, proteome, genetics, two-dimensional (2D), and three-dimensional (3D) structures. IMGT^®^ is the acknowledged high-quality integrated knowledge resource in immunogenetics for exploring immune functional genomics. IMGT^®^ comprises seven databases (for sequences, genes and 3D structures) (9– 14), 17 online tools (15– 30), and more than 15,000 pages of web resources [e.g., IMGT Scientific chart, IMGT Repertoire, IMGT Education > Aide-mémoire (31), the IMGT Medical page, the IMGT Veterinary page, the IMGT Biotechnology page, the IMGT Immunoinformatics page] (1). IMGT^®^ is the global reference in immunogenetics and immunoinformatics (32–47). Its standards have been endorsed by the World Health Organization–International Union of Immunological Societies (WHO–IUIS) Nomenclature Committee since 1995 (first IMGT^®^ online access at the Ninth International Congress of Immunology, San Francisco, CA, USA) (48, 49) and the WHO–International Nonproprietary Names (INN) Programme (50, 51).

The accuracy and the consistency of the IMGT^®^ data are based on IMGT-ONTOLOGY (52–54), the first, and so far, unique ontology for immunogenetics and immunoinformatics (8, 52–70). IMGT-ONTOLOGY manages the immunogenetics knowledge through diverse facets that rely on seven axioms: IDENTIFICATION, DESCRIPTION, CLASSIFICATION, NUMEROTATION, LOCALIZATION, ORIENTATION, and OBTENTION (53, 54, 58). The concepts generated from these axioms led to the elaboration of the IMGT^®^ standards that constitute the IMGT Scientific chart: e.g., IMGT^®^ standardized keywords (IDENTIFICATION) (59), IMGT^®^ standardized labels (DESCRIPTION) (60), IMGT^®^ standardized gene and allele nomenclature (CLASSIFICATION) (61), IMGT unique numbering (8, 62–66), and its standardized graphical 2D representation or IMGT Colliers de Perles (67–70) (NUMEROTATION).

The fundamental information generated from these IMGT-ONTOLOGY concepts, which led to the IMGT Scientific chart rules is reviewed. The major IMGT^®^ tools and databases used for IG and TR repertoire analysis, antibody humanization, and IG/Ag and TR/pMH structures are briefly presented: IMGT/V-QUEST (15–20) for the analysis of rearranged nucleotide sequence with the results of the integrated IMGT/JunctionAnalysis (21, 22), IMGT/Automat (23, 24) and IMGT/Collier-de-Perles tool (29), IMGT/HighV-QUEST, the high-throughput version for next-generation sequencing (NGS) (20, 25, 26), IMGT/DomainGapAlign (12, 27, 28) for amino acid (AA) sequence analysis, IMGT/3Dstructure-DB for 3D structures (11–13) and its extension, IMGT/2Dstructure-DB (for antibodies and other proteins for which the 3D structure is not available). IMGT^®^ tools and databases run against IMGT reference directories built from sequences annotated in IMGT/LIGM-DB (9), the IMGT^®^ nucleotide database (175,406 sequences from 346 species in November 2013) and from IMGT/GENE-DB (10), the IMGT^®^ gene database (3,117 genes and 4,732 alleles from 17 species, of which 695 genes and 1,420 alleles for *Homo sapiens* and 868 genes and 1,318 alleles for *Mus musculus* in November 2013).

An interface, IMGT/mAb-DB (14), has been developed to provide an easy access to therapeutic antibody AA sequences (links to IMGT/2Dstructure-DB) and structures (links to IMGT/3Dstructure-DB, if 3D structures are available). IMGT/mAb-DB data include monoclonal antibodies (mAb, INN suffix -mab; a -mab is defined by the presence of at least an IG variable domain) and fusion proteins for immune applications (FPIA, INN suffix -cept) (a -cept is defined by a receptor fused to an Fc) from the WHO–INN Programme (50, 51). This database also includes a few composite proteins for clinical applications (CPCA) (e.g., protein or peptide fused to an Fc for only increasing their half-life, identified by the INN prefix ef-) and some related proteins of the immune system (RPI) used, unmodified, for clinical applications. The unified IMGT^®^ approach is of major interest for bridging knowledge from IG and TR repertoire in normal and pathological situations (71–74), IG allotypes and immunogenicity (75–77), NGS repertoire (25, 26), antibody engineering, and humanization (35, 42–44, 46, 78–82).

## IMGT-Ontology Concepts

### IDENTIFICATION: IMGT^®^ standardized keywords

More than 325 IMGT^®^ standardized keywords (189 for sequences and 137 for 3D structures) were precisely defined (59). They represent the controlled vocabulary assigned during the annotation process and allow standardized search criteria for querying the IMGT^®^ databases and for the extraction of sequences and 3D structures. They have been entered in BioPortal at the National Center for Biomedical Ontology (NCBO) in 2010^2^ .

Standardized keywords are assigned at each step of the molecular synthesis of an IG. Those assigned to a nucleotide sequence are found in the “DE” (definition) and “KW” (keyword) lines of the IMGT/LIGM-DB files (9). They characterize for instance the gene type, the configuration type and the functionality type (59). There are six gene types: variable (V), diversity (D), joining (J), constant (C), conventional-with-leader, and conventional-without-leader. Four of them (V, D, J, and C) identify the IG and TR genes and are specific to immunogenetics. There are four configuration types: germline (for the V, D, and J genes before DNA rearrangement), rearranged (for the V, D, and J genes after DNA rearrangement), partially-rearranged (for D gene after only one DNA rearrangement) and undefined (for the C gene and for the conventional genes that do not rearrange). The functionality type depends on the gene configuration. The functionality type of genes in germline or undefined configuration is functional (F), open reading frame (ORF), or pseudogene (P). The functionality type of genes in rearranged or partially-rearranged configuration is either productive [no stop codon in the V–(D)–J-region and in-frame junction] or unproductive [stop codon(s) in the V–(D)–J-region, and/or out-of-frame junction].

The 20 usual AA have been classified into 11 IMGT physicochemical classes (IMGT^®^, see footnote text 1, IMGT Education > Aide-mémoire > Amino acids). The AA changes are described according to the hydropathy (3 classes), volume (5 classes), and IMGT physicochemical classes (11 classes) (31). For example, Q1 > E (+ + −) means that in the AA change (Q > E), the two AA at codon 1 belong to the same hydropathy (+) and volume (+) classes but to different IMGT physicochemical properties (−) classes (31). Four types of AA changes are identified in IMGT^®^: very similar (+ + +), similar (+ + −, + − +), dissimilar (− − +, − + −, + − −), and very dissimilar (− − −).

### DESCRIPTION: IMGT^®^ standardized labels

More than 560 IMGT^®^ standardized labels (277 for sequences and 285 for 3D structures) were precisely defined (60). They are written in capital letters (no plural) to be recognizable without creating new terms. Standardized labels assigned to the description of sequences are found in the “FT” (feature) lines of the IMGT/LIGM-DB files (9). Querying these labels represents a big plus compared to the generalist nucleotide databases [GenBank/European Nucleotide Archive (ENA)/DNA Data Bank of Japan (DDBJ)]. Thus it is possible to query for the “CDR3-IMGT” of the human rearranged productive sequences of IG-Heavy-Gamma (e.g., 1733 CDR3-IMGT obtained, with their sequences at the nucleotide or AA level). The core labels include V-REGION, D-REGION, J-REGION, and C-REGION, which correspond to the coding region of the V, D, J, and C genes. IMGT structure labels for chains and domains and their correspondence with sequence labels are shown for human IG (Table 1), for human TR (Table 2), and for MH (8) (Table 3). These labels are necessary for a standardized description of the IG, TR, and MH sequences and structures in databases and tools (60).

**Table 1:** **Immunoglobulin (IG) receptor, chain, and domain structure labels and correspondence with sequence labels**.

IG structure labels (IMGT/3Dstructure-DB)				Sequence labels (IMGT/LIGM-DB)
Receptor^a^	Chain^b^	Domain description type	Domain^c^	Region
IG-GAMMA-1_KAPPA	L-KAPPA	V-DOMAIN	V-KAPPA	V–J-REGION
		C-DOMAIN	C-KAPPA	C-REGION
	H-GAMMA-1	V-DOMAIN	VH	V–D–J-REGION
		C-DOMAIN	CH1	C-REGION^d^
		C-DOMAIN	CH2	
		C-DOMAIN	CH3	
IG-MU_LAMBDA	L-LAMBDA	V-DOMAIN	V-LAMBDA	V–J-REGION
		C-DOMAIN	C-LAMBDA-1	C-REGION
	H-MU	V-DOMAIN	VH	V–D–J-REGION
		C-DOMAIN	CH1	C-REGION^d^
		C-DOMAIN	CH2	
		C-DOMAIN	CH3
		C-DOMAIN	CH4^e^

*^a^Labels are shown for two examples of IG (*Homo sapiens* IgG1-kappa and IgM-lambda). An IG (“Receptor”) (Figure 1) is made of two identical heavy (H, for IG-HEAVY) chains and two identical light (L, for IG-LIGHT) chains (“Chain”) and usually comprises 12 (e.g., IgG1) or 14 (e.g., IgM) domains. Each chain has an N-terminal V-DOMAIN (or V–(D)–J-REGION, encoded by the rearranged V–(D)–J genes), whereas the remaining of the chain is the C-REGION (encoded by a C gene). The IG C-REGION comprises one C-DOMAIN (C-KAPPA or C-LAMBDA) for the L chain, or several C-DOMAIN (CH) for the H chain (2)*.

*^b^The kappa (L-KAPPA) or lambda (L-LAMBDA) light chains may associate to any heavy chain isotype (e.g., H-GAMMA-1, H-MU). In humans, there are nine isotypes, H-MU, H-DELTA, H-GAMMA-3, H-GAMMA-1, H-ALPHA-1, H-GAMMA-2, H-GAMMA-4, H-EPSILON, H-ALPHA-2 (listed in the order 5′–3′ in the IGH locus of the IGHC genes, which encode the constant region of the heavy chains (2) (IMGT^®^http//www.imgt.org, IMGT Repertoire)*.

*^c^The IG V-DOMAIN includes VH (for the IG heavy chain) and VL (for the IG light chain). In higher vertebrates, the VL is V-KAPPA or V-LAMBDA, whereas in fishes, the VL is V-IOTA. The C-DOMAIN includes CH [for the IG heavy chain, the number of CH per chain depending on the isotype (2)] and CL (for the IG light chain). In higher vertebrates, the CL is C-KAPPA or C-LAMBDA, whereas in fishes, the CL is C-IOTA*.

*^d^The heavy chain C-REGION also includes the HINGE-REGION for the H-ALPHA, H-DELTA, and H-GAMMA chains and, for membrane IG (mIG), the CONNECTING-REGION (CO), TRANSMEMBRANE-REGION (TM) and CYTOPLASMIC-REGION (CY); for secreted IG (sIG), the C-REGION includes CHS instead of CO, TM, and CY*.

*^e^For H-MU and H-EPSILON*.

**Table 2:** **T cell receptor (TR), chain, and domain structure labels and correspondence with sequence labels**.

TR structure labels (IMGT/3Dstructure-DB)				Sequence labels (IMGT/LIGM-DB)
Receptor^a^	Chain	Domain description type	Domain^b^	Region
TR-ALPHA_BETA	TR-ALPHA	V-DOMAIN	V-ALPHA	V–J-REGION
		C-DOMAIN	C-ALPHA	Part of C-REGION^c^
	TR-BETA	V-DOMAIN	V-BETA	V–D–J-REGION
		C-DOMAIN	C-BETA	Part of C-REGION^c^
TR-GAMMA_DELTA	TR-GAMMA	V-DOMAIN	V-GAMMA	V–J-REGION
		C-DOMAIN	C-GAMMA	Part of C-REGION^c^
	TR-DELTA	V-DOMAIN	V-DELTA	V–D–J-REGION
		C-DOMAIN	C-DELTA	Part of C-REGION^c^

*^a^A TR (“Receptor”) (3) (Figure 2) is made of two chains (alpha and beta, or gamma and delta) (“Chain”) and comprises four domains. Each chain has an N-terminal V-DOMAIN [or V–(D)–J-REGION, encoded by the rearranged V–(D)–J genes (3)] whereas the remaining of the chain is the C-REGION (encoded by a C gene). The TR C-REGION comprises one C-DOMAIN (3). TR receptor, chain, and domain structure labels, and correspondence with sequence labels, are shown for two examples of TR (*Homo sapiens* TR-alpha_beta and TR-gamma_delta)*.

*^b^The TR V-DOMAIN includes V-ALPHA, V-BETA, V-GAMMA, and V-DELTA. The TR C-DOMAIN includes C-ALPHA, C-BETA, C-GAMMA, and C-DELTA (there are two isotypes for the TR-BETA and TR-GAMMA chains in humans, TR-BETA-1 and TR-BETA-2, and TR-GAMMA-1 and TR-GAMMA-2, the C-REGION of these chains being encoded by the TRBC1 and TRBC2 genes, and TRGC1 and TRGC2 genes, respectively) (IMGT^®^http://www.imgt.org, IMGT Repertoire) (3)*.

*^c^The TR chain C-REGION also includes the CONNECTING-REGION (CO), the TRANSMEMBRANE-REGION (TM), and the CYTOPLASMIC-REGION (CY), which are not present in 3D structures*.

**Table 3:** **Major histocompatibility (MH) receptor, chain, and domain structure labels and correspondence with sequence labels**.

MH group	MH structure labels (IMGT/3Dstructure-DB)					Sequence labels (IMGT/LIGM-DB)
	Receptor^a^	Chain	Domain description type^b^	Domain	Domain number	Region
MH1	MH1-ALPHA_B2M	I-ALPHA	G-DOMAIN	G-ALPHA1	[D1]	Part of REGION^c^
			G-DOMAIN	G-ALPHA2	[D2]	
			C-LIKE-DOMAIN	C-LIKE	[D3]	
		B2M	C-LIKE-DOMAIN	C-LIKE	[D]	REGION
MH2	MH2-ALPHA_BETA	II-ALPHA	G-DOMAIN	G-ALPHA	[D1]	Part of REGION^c^
			C-LIKE-DOMAIN	C-LIKE	[D2]	
		II-BETA	G-DOMAIN	G-BETA	[D1]	Part of REGION^c^
			C-LIKE-DOMAIN	C-LIKE	[D2]	

*^a^An MH (“Receptor”) (8) depending on the MH group is made of one chain (I-ALPHA) non-covalently associated to the beta2-microglobulin (B2M) (MH1 group, in the literature MHC class I) (Figure 2) or of two chains (II-ALPHA and II-BETA) (MH2 group, in the literature MHC class II). The I-ALPHA chain has two G-DOMAIN whereas each II-ALPHA and II-BETA has one G-DOMAIN. MH receptor, chain, and domain structure labels, and correspondence with sequence labels, are shown for examples of members of the MH1 and MH2 groups*.

*^b^The domain description type shows that the MH proteins belong to the MhSF by their G-DOMAIN and to the IgSF by their C-LIKE-DOMAIN. The B2M associated to the I-ALPHA chain in MH1 has only a single C-LIKE-DOMAIN and only belongs to the IgSF*.

*^c^The REGION of the I-ALPHA, II-ALPHA, and II-BETA chains also includes the CONNECTING-REGION (CO), the TRANSMEMBRANE-REGION (TM), and the CYTOPLASMIC-REGION (CY), which are not present in 3D structures*.

Highly conserved AA at a given position in a domain have IMGT labels (60). Thus three AA labels are common to the V and C-domains: 1st-CYS (cysteine C at position 23), CONSERVED-TRP (tryptophan W at position 41), and 2nd-CYS (C at position 104) (62–66). Two other labels are characteristics of the IG and TR V-DOMAIN and correspond to the first AA of the canonical F/W–G–X–G motif (where F is phenylalanine, W tryptophan, G glycine, and X any AA) encoded by the J-REGION: J-PHE or J-TRP (F or W at position 118) (62–64, 66).

### CLASSIFICATION: IMGT^®^ standardized genes and alleles

The IMGT-ONTOLOGY CLASSIFICATION axiom was the trigger of immunoinformatics’ birth. Indeed the IMGT^®^ concepts of classification allowed, for the first time, to classify the antigen receptor genes (IG and TR) for any locus [e.g., immunoglobulin heavy (IGH), T cell receptor alpha (TRA)], for any gene configuration (germline, undefined, or rearranged), and for any species (from fishes to humans). In higher vertebrates, there are seven IG and TR major loci (other loci correspond to chromosomal orphon sets, genes of which are orphons, not used in the IG or TR chain synthesis). The IG major loci include the IGH, and for the light chains, the immunoglobulin kappa (IGK), and the immunoglobulin lambda (IGL) in higher vertebrates, and the immunoglobulin iota (IGI) in fishes (IMGT^®^, see footnote text 1, IMGT Repertoire).

Since the creation of IMGT^®^ in 1989, at New Haven during the Tenth Human Genome Mapping Workshop (HGM10), the standardized classification and nomenclature of the IG and TR of humans and other vertebrate species have been under the responsibility of the IMGT Nomenclature Committee (IMGT-NC). IMGT^®^ gene and allele names are based on the concepts of classification of “Group,” “Subgroup,” “Gene,” and “Allele” (61). “Group” allows to classify a set of genes that belong to the same multigene family, within the same species or between different species. For example, there are 10 groups for the IG of higher vertebrates: IGHV, IGHD, IGHJ, IGHC, IGKV, IGKJ, IGKC, IGLV, IGLJ, IGLC. “Subgroup” allows to identify a subset of genes, which belong to the same group, and which, in a given species, share at least 75% identity at the nucleotide level, e.g., *Homo sapiens* IGHV1 subgroup. Subgroups, genes, and alleles are always associated to a species name. An allele is a polymorphic variant of a gene, which is characterized by the mutations of its sequence at the nucleotide level, identified in its core sequence and compared to the gene allele reference sequence, designated as allele *01. For example, *Homo sapiens* IGHV1-2*01 is the allele *01 of the *Homo sapiens* IGHV1-2 gene that belongs to the *Homo sapiens* IGHV1 subgroup, which itself belongs to the IGHV group. For the IGH locus, the constant genes are designated by the letter (and eventually number) corresponding to the encoded isotypes (IGHM, IGHD, IGHG3…), instead of using the letter C. IG and TR genes and alleles are not italicized in publications. IMGT-ONTOLOGY concepts of classification have been entered in the NCBO BioPortal.

The IMGT^®^ IG and TR gene names (2–5) were approved by the Human Genome Organisation (HUGO) Nomenclature Committee (HGNC) in 1999 (83, 84) and were endorsed by the WHO–IUIS Nomenclature Subcommittee for IG and TR (48, 49). The IMGT^®^ IG and TR gene names are the official international reference and, as such, have been entered in IMGT/GENE-DB (10), in the Genome Database (GDB) (85), in LocusLink at the National Center for Biotechnology Information (NCBI) USA (86), in Entrez Gene (NCBI) when this database (now designated as “Gene”) superseded LocusLink (87), in NCBI MapViewer, in Ensembl at the European Bioinformatics Institute (EBI) (88), and in the Vertebrate Genome Annotation (Vega) Browser (89) at the Wellcome Trust Sanger Institute (UK). HGNC, Gene NCBI, Ensembl, and Vega have direct links to IMGT/GENE-DB (10). IMGT^®^ human IG and TR genes were also integrated in IMGT-ONTOLOGY on the NCBO BioPortal and, on the same site, in the HUGO ontology and in the National Cancer Institute (NCI) Metathesaurus. AA sequences of human IG and TR constant genes (e.g., *Homo sapiens* IGHM, IGHG1, IGHG2) were provided to UniProt in 2008. Since 2007, IMGT^®^ gene and allele names have been used for the description of the therapeutic mAb and FPIA of the WHO–INN Programme (50, 51).

The basis for the nomenclature of the MH of newly sequenced genomes has been set up on the same concepts. In IMGT^®^, MHC refers to the locus, which indeed is a complex of genes, particularly in the higher vertebrates. In contrast the letter “C” is dropped when referring to individual genes and proteins. Thus, the class I genes are designated as MH1 whereas the class II genes are designated as MH2. The IMGT nomenclature, with the MH1 and MH2 groups, has been used for the first time with the *Oncorhynchus mykiss* genes [see footnote text 1, IMGT Repertoire (MH) > Locus and genes > Gene tables]. It can also be applied to the human genes in databases, which deal with humans and other vertebrate species (for example, *Homo sapiens* MH1-A for HLA-A).

### NUMEROTATION: IMGT unique numbering and IMGT Collier de Perles

The IMGT-ONTOLOGY NUMEROTATION axiom is acknowledged as the “IMGT^®^ Rosetta stone” that has bridged the biological and computational spheres in bioinformatics (40). The IMGT^®^ concepts of numerotation comprise the IMGT unique numbering (8, 62–66) and its graphical 2D representation the IMGT Collier de Perles (67–70). Developed for and by the “domain,” these concepts integrate sequences, structures, and interactions into a standardized domain-centric knowledge for functional genomics. The IMGT unique numbering has been defined for the variable V-domain (V-DOMAIN of the IG and TR, and V-LIKE-DOMAIN of IgSF other than IG and TR) (62–64), the constant C-domain (C-DOMAIN of the IG and TR, and C-LIKE-DOMAIN of IgSF other than IG and TR) (65), and the groove G-domain (G-DOMAIN of the MH, and G-LIKE-DOMAIN of MhSF other than MH) (8, 90, 91). Thus the IMGT unique numbering and IMGT Collier de Perles provide a definitive and universal system across species including invertebrates, for the sequences and structures of the V, C, and G-domains of IG, TR, MH, IgSF, and MhSF (66, 70, 92, 93).

#### V-domain IMGT^®^ definitive system

##### V-domain definition and main characteristics

In the IMGT^®^ definitive system, the V-domain includes the V-DOMAIN of the IG and of the TR, which corresponds to the V–J-REGION or V–D–J-REGION encoded by V–(D)–J rearrangements (2, 3), and the V-LIKE-DOMAIN of the IgSF other than IG and TR. The V-domain description of any receptor, any chain, and any species is based on the IMGT unique numbering for V-domain (V-DOMAIN and V-LIKE-DOMAIN) (62–64, 66).

**Figure 3:**
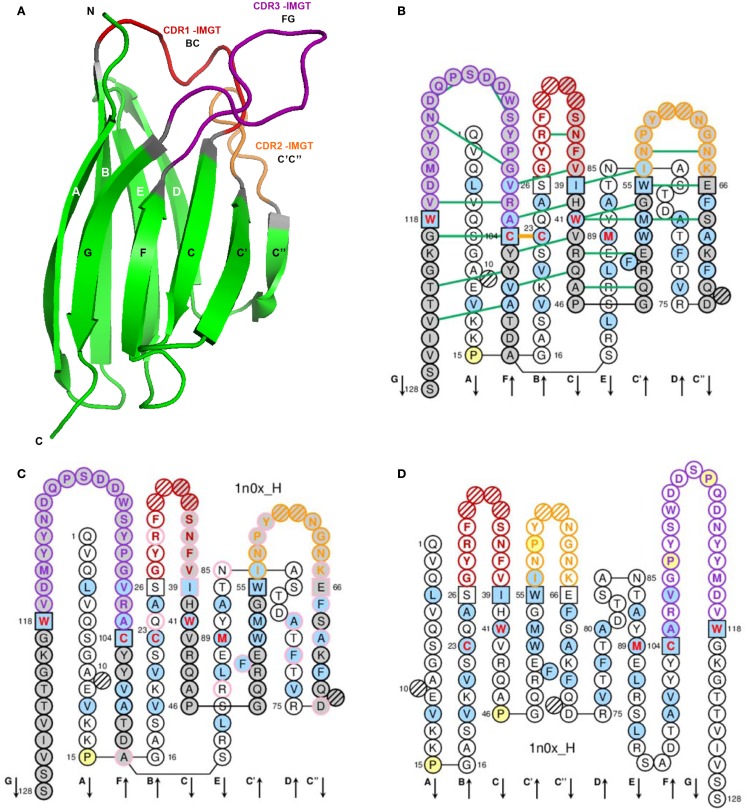
**Variable (V) domain**. An IG VH (V-DOMAIN) is shown as an example. **(A)** 3D structure ribbon representation with the IMGT strand and loop delimitations (64). **(B)** IMGT Collier de Perles on two layers with hydrogen bonds. The IMGT Collier de Perles on two layers show, in the forefront, the GFCC’C” strands (forming the sheet located at the interface VH/VL of the IG) and, in the back, the ABED strands. The IMGT Collier de Perles with hydrogen bonds (green lines online, only shown here for the GFCC′C″ sheet) is generated by the IMGT/Collier-de-Perles tool integrated in IMGT/3Dstructure-DB, from experimental 3D structure data (11–13). **(C)** IMGT Collier de Perles on two layers generated from IMGT/DomainGapAlign (12, 27, 28). Pink circles (online) indicate amino acid (AA) changes compared to the closest genes and alleles from the IMGT reference directory. **(D)** IMGT Collier de Perles on one layer. AA are shown in the one-letter abbreviation. All proline (P) are shown online in yellow. IMGT anchors are in square. Hatched circles are IMGT gaps according to the IMGT unique numbering for V-domain (64, 66). Positions with bold (online red) letters indicate the four conserved positions that are common to a V-domain and to a C-domain: 23 (1st-CYS), 41 (CONSERVED-TRP), 89 (hydrophobic), 104 (2nd-CYS) (62–66), and the fifth conserved position, 118 (J-TRP or J-PHE), which is specific to a V-DOMAIN and belongs to the motif F/W-G-X-G that characterizes the J-REGION (64, 66) (Table 4). The hydrophobic AA (hydropathy index with positive value: I, V, L, F, C, M, A) and tryptophan (W) (31) found at a given position in more than 50% of sequences are shown (online with a blue background color). Arrows indicate the direction of the beta strands and their designations in 3D structures. IMGT color menu for the CDR-IMGT of a V-DOMAIN indicates the type of rearrangement, V–D–J (for a VH here, red, orange, and purple) or V–J (for V-KAPPA or V-LAMBDA (not shown), blue, green, and greenblue) (2). The identifier of the chain to which the VH domain belongs is 1n0x_H (from the *Homo sapiens* b12 Fab) in IMGT/3Dstructure-DB (http://www.imgt.org). The CDR-IMGT lengths of this VH are [8.8.20] and the FR-IMGT are [25.17.38.11]. The 3D ribbon representation was obtained using PyMOL (http://www.pymol.org) and “IMGT numbering comparison” of 1n0x_H (VH) from IMGT/3Dstructure-DB (http://www.imgt.org).

A V-domain (Figure 3) comprises about 100 AA and is made of nine antiparallel beta strands (A, B, C, C′, C″, D, E, F, and G) linked by beta turns (AB, CC′, C″D, DE, and EF), and three loops (BC, C′C″, and FG), forming a sandwich of two sheets [ABED] [GFCC′C″] (62–64, 66). The sheets are closely packed against each other through hydrophobic interactions giving a hydrophobic core, and joined together by a disulfide bridge between a first highly conserved cysteine (1st-CYS) in the B strand (in the first sheet) and a second equally conserved cysteine (2nd-CYS) in the F strand (in the second sheet) (62–64, 66).

##### V-domain strands and loops (FR-IMGT and CDR-IMGT)

The V-domain strands and loops and their delimitations and lengths, based on the IMGT unique numbering for V-domain (62–64, 66), are shown in Table 4. In the IG and TR V-DOMAIN, the three hypervariable loops BC, C′C″, and FG involved in the ligand recognition (native antigen for IG and pMH for TR) are designated complementarity determining regions (CDR-IMGT), whereas the strands form the framework region (FR-IMGT), which includes FR1-IMGT, FR2-IMGT, FR3-IMGT, and FR4-IMGT (Table 4). In the IMGT^®^ definitive system, the CDR-IMGT have accurate and unambiguous delimitations in contrast to the CDR described in the literature. Correspondences between the IMGT unique numbering with other numberings, e.g., Kabat (94) or Chothia (95), are available in the IMGT Scientific chart. The correspondences with these previous and heterogenous numberings are useful for the interpretation of previously published data but nowadays the usage of these numberings has become obsolete owing to the development of immunoinformatics based on the IMGT^®^ standards (8, 62–70) (IMGT^®^, see footnote text 1, IMGT Scientific chart > Numbering > Correspondence between V numberings).

**Table 4:** **V-domain strands and loops, IMGT positions, and lengths, based on the IMGT unique numbering for V-domain (V-DOMAIN and V-LIKE-DOMAIN)**.

V-domain strands and loops^a^	IMGT positions^b^	Lengths^c^	Characteristic IMGT Residue@Position^d^	V-DOMAIN FR-IMGT and CDR-IMGT
A-STRAND	1–15	15 (14 if gap at 10)		FR1-IMGT
B-STRAND	16–26	11	1st-CYS 23	
BC-LOOP	27–38	12 (or less)		CDR1-IMGT
C-STRAND	39–46	8	CONSERVED-TRP 41	FR2-IMGT
C′-STRAND	47–55	9		
C′C″-LOOP	56–65	10 (or less)		CDR2-IMGT
C″-STRAND	66–74	9 (or 8 if gap at 73)		FR3-IMGT
D-STRAND	75–84	10 (or 8 if gaps at 81, 82)		
E-STRAND	85–96	12	Hydrophobic 89	
F-STRAND	97–104	8	2nd-CYS 104	
FG-LOOP	105–117	13 (or less, or more)		CDR3-IMGT
G-STRAND	118–128	11 (or 10)	V-DOMAIN J-PHE 118 or J-TRP 118^e^	FR4-IMGT

*^a^IMGT^®^ labels (concepts of description) are written in capital letters (no plural) (60). Beta turns (AB, CC′, C″D, DE, or EF) are individualized only if they have additional AA compared to the standard description. If not, they are included in the strands*.

*^b^Based on the IMGT unique numbering for V-domain (V-DOMAIN and V-LIKE-DOMAIN) (62–64, 66)*.

*^c^In number of AA (or codons)*.

*^d^IMGT Residue@Position is a given residue (usually an AA) or a given conserved property AA class, at a given position in a domain, based on the IMGT unique numbering (66)*.

*^e^In the IG and TR V-DOMAIN, the G-STRAND (or FR4-IMGT) is the C-terminal part of the J-REGION, with J-PHE or J-TRP 118, and the canonical motif F/W–G–X–G at positions 118–121 (2, 3). The JUNCTION refers to the CDR3-IMGT plus the two anchors 2nd-CYS 104 and J-PHE or J-TRP 118 (63, 64). The JUNCTION (positions 104–118) is therefore two AA longer than the corresponding CDR3-IMGT (positions 105–117) (63, 64)*.

For a V-domain, the BC loop (or CDR1-IMGT in a V-DOMAIN) encompasses positions 27–38, the C′C″ loop (or CDR2-IMGT in a V-DOMAIN) positions 56–65, and the FG loop (or CDR3-IMGT) positions 105–117. In a V-DOMAIN, the CDR3-IMGT encompasses the V–(D)–J junction that results from a V–J or V–D–J rearrangement (2, 3) and is more variable in sequence and length than the CDR1-IMGT and CDR2-IMGT that are encoded by the V gene region only. For CDR3-IMGT of length >13 AA, additional IMGT positions are added at the top of the loop between 111 and 112 (Table 5).

**Table 5:** **IMGT additional positions for CDR3-IMGT**.

CDR3-IMGT lengths	IMGT additional positions for CDR3-IMGT length >13 AA^a^									
21	111	111.1	111.2	111.3	111.4	112.4	112.3	112.2	112.1	112
20	111	111.1	111.2	111.3	–	112.4	112.3	112.2	112.1	112
19	111	111.1	111.2	111.3	–	–	112.3	112.2	112.1	112
18	111	111.1	111.2	–	–	–	112.3	112.2	112.1	112
17	111	111.1	111.2	–	–	–	–	112.2	112.1	112
16	111	111.1	–	–	–	–	–	112.2	112.1	112
15	111	111.1	–	–	–	–	–	–	112.1	112
14	111	–	–	–	–	–	–	–	112.1	112

*^a^For CDR3-IMGT length >13 AA, IMGT additional positions are created between positions 111 and 112 at the top of the CDR3-IMGT loop in the following order 112.1, 111.1, 112.2, 111.2, 112.3, 111.3, etc., and as many positions can be added as necessary for very long CDR3-IMGT. For CDR3-IMGT length <13 AA (not shown), IMGT gaps are created classically from the top of the loop, in the following order 111, 112, 110, 113, 109, 114, etc. (IMGT^®^http://www.imgt.org, IMGT Scientific chart > Numbering)*.

##### IMGT Colliers de Perles

The loop and strands are visualized in the IMGT Colliers de Perles (67– 70), which can be displayed on one layer (closer to the AA sequence) or on two layers (closer to the 3D structure) (Figure 3). The three loops, BC, C′C″, and FG (or CDR1-IMGT, CDR2-IMGT, and CDR3-IMGT for a V-DOMAIN) are delimited by the IMGT anchors, which are shown in square in the IMGT Colliers de Perles. IMGT anchors are positions, which belong to strands and represent anchors for the loops of the V-domains. IMGT anchors are the key and original concept of IMGT^®^, which definitively solved the ambiguous situation of different CDR lengths and delimitations found in the literature. The six anchors of a V-domain are positions 26 and 39 (anchors of the BC loop or CDR1-IMGT in V-DOMAIN), 55 and 66 (anchors of the C′–C″ loop or CDR2-IMGT in V-DOMAIN), 104 and 118 (anchors of the FG loop or CDR3-IMGT in V-DOMAIN). The CDR3-IMGT anchors are highly conserved, they are C104 (2nd-CYS, in F strand) and F118 or W118 (J-PHE or J-TRP in G strand). The JUNCTION of an IG or TR V-DOMAIN includes the anchors 104 and 118, and is therefore two AA longer than the corresponding CDR3-IMGT (positions 105–117).

In biological data, the lengths of the loops and strands are given by the number of occupied positions [unoccupied positions or “IMGT gaps” are represented with hatches in the IMGT Colliers de Perles (Figure 3) or by dots in alignments]. The CDR-IMGT lengths are given in number of AA (or codons), into brackets and separated by dots: for example [9.6.9] means that the BC, C′C″, and FG loops (or CDR1-IMGT, CDR2-IMGT, and CDR3-IMGT for a V-DOMAIN) have a length of 9, 6, and 9 AA (or codons), respectively. Similarly [25.17.38.11] means that the FR1-IMGT, FR2-IMGT, FR3-IMGT, and FR4-IMGT have a length of 25, 17, 38, and 11 AA (or codons), respectively. Together, the four FR of a VH domain usually comprise 91 AA and the individual FR-IMGT lengths are [25.17.38.11], whereas the four FR of a VL domain usually comprise 89 AA and the individual FR-IMGT lengths are [26.17.36.10].

##### Conserved AA

A V-domain has five characteristic AA at given positions (positions with bold (online red) letters in the IMGT Colliers de Perles). Four of them are highly conserved and hydrophobic (31) and are common to the C-domain: 23 (1st-CYS), 41 (CONSERVED-TRP), 89 (hydrophobic), and 104 (2nd-CYS). These AA contribute to the two major features shared by the V and C-domain: the disulfide bridge (between the two cysteines 23 and 104) and the internal hydrophobic core of the domain (with the side chains of tryptophan W41 and AA 89). The fifth position, 118, is an anchor of the FG loop. It is occupied, in the V-domains of IgSF other than IG or TR, by AA with diverse physicochemical properties (31). In contrast, in IG and TR V-DOMAIN, the position 118 is occupied by remarkably conserved AA, which consist in a phenylalanine or a tryptophan encoded by the J-REGION and therefore designated J-TRP or J-PHE 118. The bulky aromatic side chains of J-TRP and J-PHE are internally orientated and structurally contribute to the V-DOMAIN hydrophobic core (64).

##### Genomic delimitation

A last criterion used in the IMGT^®^ definitive system for the characterization of a V-domain is its delimitation taking into account the exon delimitations, whenever appropriate. The exon rule is not used for the delimitation of the 5′ end of the first N-terminal domain of proteins with a leader (this includes the V-DOMAIN of the IG and TR chains). In those cases, the 5′end of the first N-terminal domain of the chain corresponds to the proteolytic site between the leader (L-REGION) and the coding region of the mature protein. The IG and TR V-DOMAIN is therefore delimited in 5′ by a proteolytic site and in 3′ at the genomic level by the splicing site of the J-REGION (60). This IMGT^®^ genomic approach integrates the strands A and G, in contrast to structural alignments that usually lack these strands due to their poor structural conservation, and thus bridges the gap between genomic data (exon) and 3D structure (domain).

#### C-domain IMGT^®^ definitive system

##### C-domain definition and main characteristics

In the IMGT^®^ definitive system, the C-domain includes the C-DOMAIN of the IG and of the TR (2, 3) and the C-LIKE-DOMAIN of the IgSF other than IG and TR. The C-domain description of any receptor, any chain, and any species is based on the IMGT unique numbering for C-domain (C-DOMAIN and C-LIKE-DOMAIN) (65, 66).

**Figure 4:**
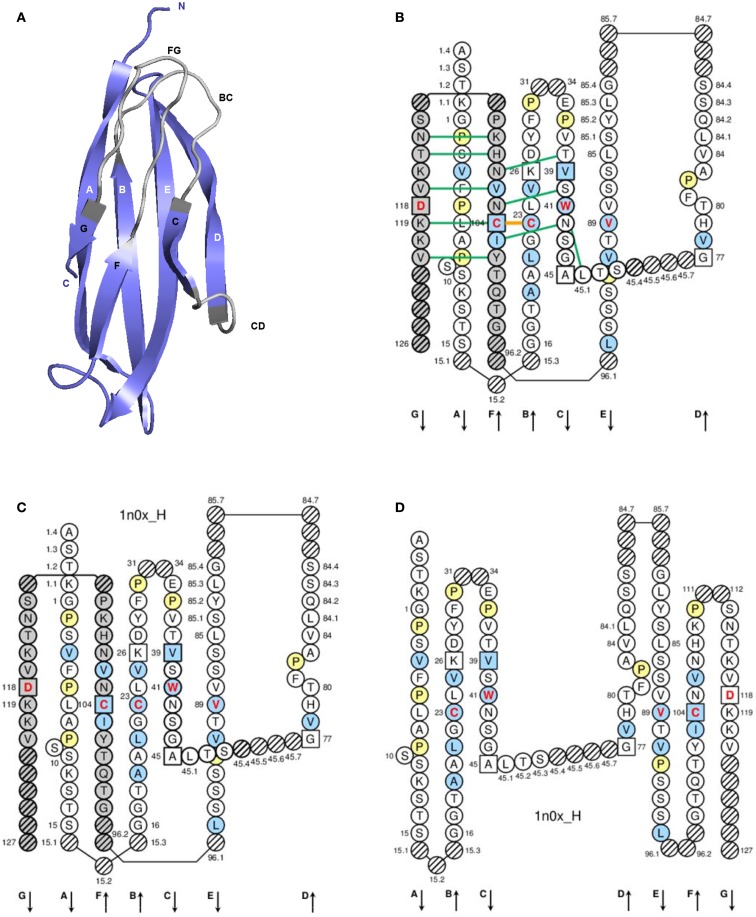
**Constant (C) domain**. An IG CH (C-DOMAIN) is shown as an example. **(A)** 3D structure ribbon representation with the IMGT strand and loop delimitations (65). **(B)** IMGT Collier de Perles on two layers with hydrogen bonds. The IMGT Colliers de Perles on two layers show, in the forefront, the GFC strands and, in the back, the ABED strands (located at the interface CH1/CL of the IG), linked by the CD transverse strand. The IMGT Collier de Perles with hydrogen bonds (green lines online, only shown here for the GFC sheet) is generated by the IMGT/Collier-de-Perles tool integrated in IMGT/3Dstructure-DB, from experimental 3D structure data (11–13). **(C)** IMGT Collier de Perles on two layers from IMGT/DomainGapAlign (12, 27, 28). **(D)** IMGT Colliers de Perles on one layer. Amino acids are shown in the one-letter abbreviation. All proline (P) are shown online in yellow. IMGT anchors are in square. Hatched circles are IMGT gaps according to the IMGT unique numbering for C-domain (65, 66). Positions with bold (online red) letters indicate the four conserved positions that are common to a V-domain and to a C-domain: 23 (1st-CYS), 41 (CONSERVED-TRP), 89 (hydrophobic), 104 (2nd-CYS) (62–66) (Table 6), and position 118, which is only conserved in V-DOMAIN. The identifier of the chain to which the CH domain belongs is 1n0x_H (from the *Homo sapiens* b12 Fab, in IMGT/3Dstructure-DB, http://www.imgt.org). The 3D ribbon representation was obtained using PyMOL and “IMGT numbering comparison” of 1n0x_H (CH1) from IMGT/3Dstructure-DB (http://www.imgt.org).

A C-domain (Figure 4) comprises about 90–100 AA and is made of seven antiparallel beta strands (A, B, C, D, E, F, and G), linked by beta turns (AB, DE, and EF), a transverse strand (CD) and two loops (BC and FG), and forming a sandwich of two sheets (ABED) (GFC) (65, 66). A C-domain has a topology and a three-dimensional structure similar to that of a V-domain but without the C′ and C″ strands and the C′C″ loop, which is replaced by a transverse CD strand (65).

##### C-domain strands and loops

The C-domain strands, turns, and loops and their delimitations and lengths, based on the IMGT unique numbering for C-domain (65, 66), are shown in Table 6. Correspondences between the IMGT unique numbering with other numberings (Eu, Kabat) are available in the IMGT Scientific chart. The correspondences with these previous numberings are useful for the interpretation of previously published data but, as for the V-domain, the usage of these previous numberings has become obsolete owing to the development of immunoinformatics based on the IMGT^®^ standards (8, 62–70) (IMGT^®^, see footnote text 1, IMGT Scientific chart > Numbering > Correspondence between C numberings).

**Table 6:** **C-domain strands, turns, and loops, IMGT positions, and lengths, based on the IMGT unique numbering for C-domain (C-DOMAIN and C-LIKE-DOMAIN)**.

C-domain strands, turns, and loops^a^	IMGT positions^b^	Lengths^c^	Characteristic IMGT Residue@Position^d^
A-STRAND	1–15	15 (14 if gap at 10)	
AB-TURN	15.1–15.3	0–3	
B-STRAND	16–26	11	1st-CYS 23
BC-LOOP	27–31	10 (or less)	
	34–38	
C-STRAND	39–45	7	CONSERVED-TRP 41
CD-STRAND	45.1–45.9	0–9	
D-STRAND	77–84	8 (or 7 if gap at 82)	
DE-TURN	84.1–84.7	0–14	
	85.1–85.7	
E-STRAND	85–96	12	Hydrophobic 89
EF-TURN	96.1–96.2	0–2	
F-STRAND	97–104	8	2nd-CYS 104
FG-LOOP	105–117	13 (or less, or more)	
G-STRAND	118–128	11 (or less)	

*^a^IMGT^®^ labels (concepts of description) are written in capital letters (no plural) (60)*.

*^b^Based on the IMGT unique numbering for C-domain (C-DOMAIN and C-LIKE-DOMAIN) (65, 66)*.

*^c^In number of amino acids (AA) (or codons)*.

*^d^IMGT Residue@Position is a given residue (usually an AA) or a given conserved property AA class, at a given position in a domain, based on the IMGT unique numbering (66)*.

##### IMGT Colliers de Perles

The lengths of the strands and loops are visualized in the IMGT Colliers de Perles (68–70), on one layer and two layers (Figure 4). There are six IMGT anchors in a C-domain (four of them identical to those of a V-domain): positions 26 and 39 (anchors of the BC loop), 45 and 77 [by extension, anchors of the CD strand as there is no C′–C″ loop in a C-domain (65)], and 104 and 118 (anchors of the FG loop).

##### Conserved AA

A C-domain has five characteristic AA at given positions [positions with bold (online red) letters in the IMGT Colliers de Perles]. Four of them are highly conserved and hydrophobic (31) and are common to the V-domain: 23 (1st-CYS), 41 (CONSERVED-TRP), 89 (hydrophobic), and 104 (2nd-CYS). As mentioned above, these AA contribute to the two major features shared by the V and C-domain: the disulfide bridge (between the two cysteines 23 and 104) and the internal hydrophobic core of the domain (with the side chains of tryptophan W41 and AA 89). The fifth position, 118, is diverse and is characterized as being an FG loop anchor.

##### Genomic delimitation

In the IMGT^®^ definitive system, the C-domains (C-DOMAIN and C-LIKE-DOMAIN) are delimited taking into account the exon delimitation, whenever appropriate. As for the V-domain, this IMGT^®^ genomic approach integrates the strands A and G, which are absent of structural alignments.

#### G-domain IMGT^®^ definitive system

##### G-domain definition and main characteristics

**Figure 5:**
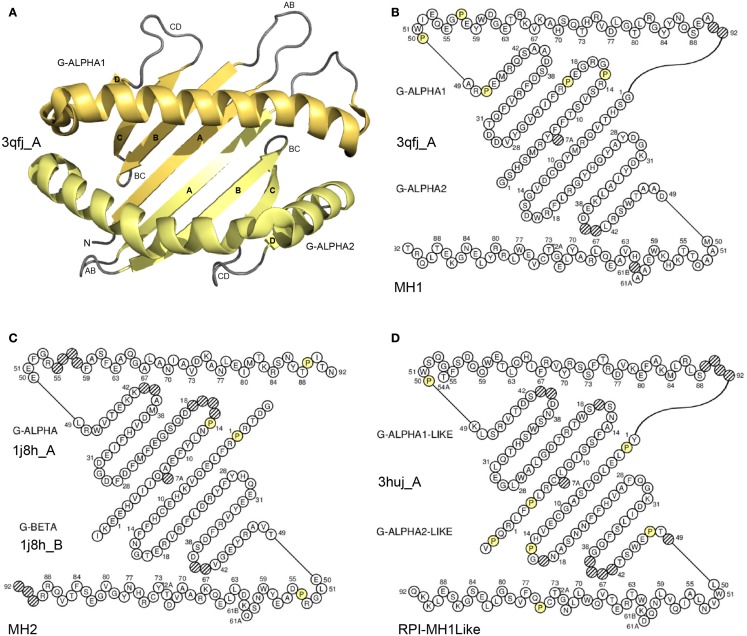
**Groove (G) domain**. **(A)** 3D structure ribbon representation of the two G-domains. The two domains form a groove with a “floor” (four strands from each domain) and two “walls” (one helix from each domain) (8). The G-domains characterize the proteins of the MhSF, which comprises the MH (MH1 and MH2) and the RPI-MH1Like (MhSF other than MH) (8). The two G-DOMAIN of an MH1 are shown as an example. The view is from above the cleft with the G-ALPHA1 (on top) and G-ALPHA2 (on bottom). **(B)** IMGT Colliers de Perles of the two G-DOMAIN of an MH1. G-ALPHA1 (on top) and G-ALPHA2 (on bottom) belong to the I-ALPHA chain (8). **(C)** IMGT Colliers de Perles of the two G-DOMAIN of an MH2. G-ALPHA (on top) and G-BETA (on bottom) to the II-ALPHA chain and to the II-BETA chain, respectively (8). **(D)** IMGT Colliers de Perles of the two G-LIKE-DOMAIN of a RPI-MH1Like. G-ALPHA1-LIKE (on top) and G-ALPHA2-LIKE (on bottom) belong to the I-ALPHA-LIKE chain. Helices are moved outside of the floor to make it visible. Amino acids (AA) are shown in the one-letter abbreviation. All proline (P) are shown online in yellow. Hatched circles are IMGT gaps according to the IMGT unique numbering for G-domain (8, 66). The 3D ribbon representation was obtained using PyMOL and “IMGT numbering comparison” of 3qfj_A (G-ALPHA1 and G-ALPHA2) in IMGT/3Dstructure-DB (http://www.imgt.org). IMGT Colliers de Perles AA sequences are from 3qfj_A for MH1 (*Homo sapiens* HLA-A*0201), 1j8h_A and 1j8h_B for MH2 (*Homo sapiens* HLA-DRA*0101 and HLA-DRB1*0401, respectively) and 3huj_A for RPI-MH1Like (*Homo sapiens* CD1D). The IMGT Colliers de Perles were generated using the IMGT/Collier-de-Perles tool integrated in IMGT/3Dstructure-DB (http://www.imgt.org) (11–13).

In the IMGT^®^ definitive system, the G-domain includes the G-DOMAIN of the MH (Figure 5) (8, 66) and the G-LIKE-DOMAIN of the MhSF other than MH or RP1-MH1Like (there is no “RPI-MH2Like” identified so far) (96, 97). The RPI-MH1Like in humans comprise (97): AZGP1 (that regulates fat degradation in adipocytes), CD1A to CD1E proteins (that display phospholipid antigens to T cells and participate in immune defense against microbian pathogens), FCGRT (that transports maternal immunoglobulins through placenta and governs neonatal immunity), HFE (that interacts with transferring receptor and takes part in iron homeostasis by regulating iron transport through cellular membranes), MICA and MICB (that are induced by stress and involved in tumor cell detection), MR1 (that regulates mucosal immunity), PROCR, previously EPCR (that interacts with activated C protein and is involved in the blood coagulation pathway), RAET1E, RAET1G, and RAET1L (that are inducible by retinoic acid and stimulate cytokine/chemokine production and cytotoxic activity of NK cells), and ULBP1, ULBP2, and ULBP3 (that are ligands for NKG2D receptor). The G-domain description of any receptor, any chain, and any species is based on the IMGT unique numbering for G-domain (G-DOMAIN and G-LIKE-DOMAIN) (8, 66).

##### G-domain strands and helix

A G-domain (Figure 5) comprises about 90 AA and is made of four antiparallel beta strands (A, B, C, and D) linked by turns (AB, BC, and CD), and of a helix (98, 99); the helix sits on the beta strands, its axis forming an angle of about 40° with the strands (90, 91). Two G-domains are needed to form the MhSF groove made of a “floor” and two “walls” (8, 66). Each G-domain contributes by its four strands and turns to half of the groove floor and by its helix to one wall of the groove (8, 66, 90, 91). The MH groove in which the peptide binds is made of two G-DOMAIN belonging to a single chain or to two chains, depending on the MH group, MH1 or MH2, respectively. In the MH1, the groove is made of two G-DOMAIN (G-ALPHA1 and G-ALPHA2), which belong to the same chain I-ALPHA, whereas in the MH2, the groove is made of two G-DOMAIN (G-ALPHA and G-BETA), which belong to two different chains, II-ALPHA and II-BETA, respectively (8, 66). For the RPI-MH1Like, the two G-LIKE-DOMAIN also belong, as for the MH1, to the same chain (I-ALPHA-LIKE) (96, 97).

##### IMGT Colliers de Perles

The G-domain strands, turns, and helix and their delimitations and lengths, based on the IMGT unique numbering for G-domain (8, 66) are shown in Table 7. The strands and helix of each domain are visualized in the IMGT Collier de Perles (68–70, 90, 91) (Figure 5). The views are from above the cleft (with the helices displaced to show the floor) and with on top and on bottom, respectively, G-ALPHA1 and G-ALPHA2 (MH1), G-ALPHA and G-BETA (MH2), and G-ALPHA1-LIKE and G-ALPHA2-LIKE (RPI-MH1Like). There is no link between G-ALPHA and G-BETA because they belong to different chains (II-ALPHA and II-BETA).

**Table 7:** **G-domain strands, turns, and helix, IMGT positions, and lengths, based on the IMGT unique numbering for G-domain (G-DOMAIN and G-LIKE-DOMAIN)**.

G-domain strands, turns, and helix^a^	IMGT positions^b^	Lengths^c^	Characteristic IMGT Residue@Position^d^ and additional positions^e^
A-STRAND	1–14	14	7A, CYS-11
AB-TURN	15–17	3 (or 2 or 0)	
B-STRAND	18–28	11 (or 10^f^)	
BC-TURN	29–30	2	
C-STRAND	31–38	8	
CD-TURN	39–41	3 (or 1^g^)	
D-STRAND	42–49	8	49.1–49.5
HELIX	50–92	43 (or less or more)	54A, 61A, 61B, 72A, CYS-74, 92A

*^a^IMGT^®^ labels (concepts of description) are written in capital letters (no plural) (60)*.

*^b^Based on the IMGT unique numbering for G-domain (G-DOMAIN and G-LIKE-DOMAIN) (8, 66)*.

*^c^In number of AA (or codons)*.

*^d^IMGT Residue@Position is a given residue (usually an AA) or a given conserved property AA class, at a given position in a domain, based on the IMGT unique numbering (66)*.

*^e^For details on additional positions, see Ref. (8)*.

*^f^Or 9 in some G-BETA (8)*.

*^g^Or 0 in some G-ALPHA2-LIKE (8)*.

##### Conserved AA

Two conserved cysteines, CYS-11 (in the A-strand) and CYS-74 (in the helix) (Table 7), are found in the G-ALPHA2, G-BETA, and G-ALPHA2-LIKE (Figure 5), where they form a disulfide bridge fixing the helix to the floor.

##### Genomic delimitation

In the IMGT^®^ definitive system, the G-domains (G-DOMAIN and G-LIKE-DOMAIN) are delimited taking into account the exon delimitations, if appropriate. Alignment sequence comparison with previously identified genes is used when genomic data are not available, as recently done for the rainbow trout (*Oncorhynchus mykiss*) MH1 and MH2 [IMGT^®^, see footnote text 1, IMGT Repertoire (MH) > Proteins and alleles > Protein displays].

#### IMGT/Collier-de-Perles tool

The IMGT/Collier-de-Perles tool (29), on the IMGT^®^ Web site at http://www.imgt.org, is a generic tool, which allows the users to draw IMGT Colliers de Perles (67–70) starting from their own domain AA sequences [sequences already gapped according to the IMGT unique numbering, using for example IMGT/DomainGapAlign (12, 27, 28)] (Table 8). IMGT Collier de Perles can be obtained for V and C-domains (on one or two layers) and for G-domains (with one or the two domains of the groove). IMGT/Collier-de-Perles tool online can be customized to display the IG and TR CDR-IMGT according to the IMGT color menu and the AA according to their hydropathy or volume, or to the 11 IMGT physicochemical classes (31).

**Table 8:** **IMGT^®^ tools and databases for the analysis of the IG, TR, and MH domains (http://www.imgt.org)**.

IMGT^®^ tools	Results for V, C, or G-domains^a^	Entry types and protocol references
IMGT/Collier-de-Perles (29)	Graphical 2D representation of IMGT Colliers de Perles (67–70)	User “IMGT gapped” V, C, or G-domain amino acid (AA) sequences (one sequence per representation, two possible for G) (29)
**IG AND TR REPERTOIRE ANALYSIS**		
IMGT/V-QUEST (15–20)	1. Introduction of IMGT gaps	User nucleotide sequences of V-DOMAIN (1–50 sequences per analysis, and 1–10 sequences with the option “Search for insertions and deletions”) (19)
	2. Identification of the closest V, D, and J genes and alleles	
	3. IMGT/JunctionAnalysis results (21, 22)	
	4. Description of mutations and AA changes	
		*Applications:* somatic mutations in chronic lymphocytic leukemia (CLL) prognostic
	5. Identification of indels and their correction (19) (option)	
	6. IMGT/Automat annotation (23, 24)	
	7. IMGT Colliers de Perles (29)	
IMGT/HighV-QUEST (20, 25, 26)	1. Introduction of IMGT gaps	User NGS long (e.g., from 454) nucleotide sequences of V-DOMAIN (up to 500,000 sequences per run^b^)^c^ (25, 26)
	2. Identification of indels and their correction (19) (by default)	
	3. Identification of the closest V, D, and J genes and alleles	
		*Applications:* IG and TR immune repertoires and clonotypes in NGS
	4. IMGT/JunctionAnalysis results (21, 22)	
	5. Description of mutations and AA changes	
	6. IMGT/Automat annotation (23, 24)	
	7. Statistical analysis (25)	
	8. Characterization of the IMGT clonotypes (AA) (26)	
**IG, TR, MH DOMAIN AA SEQUENCE ANALYSIS**		
IMGT/DomainGapAlign (12, 27, 28)	1. Introduction of IMGT gaps	User AA sequences of V, C, and G-domains (one to several sequences of same domain type) (27, 28)
	2. Identification of the closest genes and alleles	
	3. Delimitation of the domains	
	4. Description of AA changes	*Applications:* IMGT antibody engineering and humanization for V and C
	5. IMGT Colliers de Perles (67–70) with highlighted AA changes (pink circles online)	
**IMGT^®^ DATABASES**		
IMGT/3Dstructure-DB (11–13)	1. Identification of the closest genes and alleles	2,290 structure entries (1,987 IG, including 852 IG/Ag complexes, 151 TR, and 542 MH including 84 TR/pMH complexes)^b^
	2. IMGT/DomainGapAlign results (12, 27, 28)	
	3. IMGT Collier de Perles (67–70) (on two layers with hydrogen bonds for V and C or with pMH contact sites for G)	
		*Applications:* identification of the paratope and epitope in IG/AG and TR/pMH complexes and pMH contacts
	4. Contact analysis between a pair of domains or between a domain and a ligand	
	5. Renumbered IMGT files	
	6. IMGT numbering comparison	
IMGT/2Dstructure-DB (13)*	1. Identification of the closest genes and alleles	512 AA sequence entries^b^ (of which 506 IG)*
	2. IMGT/DomainGapAlign results (12, 27, 28)	
		*Applications:* from gene to structures in the absence of 3D
	3. IMGT Collier de Perles (67–70)	
	4. Renumbered IMGT files	

An asterisk (*) indicates that parts of the protocol dealing with 3D structures (hydrogen bonds in IMGT Colliers de Perles on two layers, Contact analysis) are not relevant, otherwise all other queries and results are similar to IMGT/3Dstructure-DB.

*^a^V: V-domain (includes V-DOMAIN of IG and TR and V-LIKE-DOMAIN of IgSF other than IG and TR) (64). C: C-domain (includes C-DOMAIN of IG and TR and C-LIKE-DOMAIN of IgSF other than IG and TR) (65). G: G-domain (G-DOMAIN of MH and G-LIKE-DOMAIN of MhSF other than MH) (8)*.

*^b^In November 2013*.

*^c^In November 2013, more than 1.4 billions of sequences analyzed by IMGT/HighV-QUEST, by 702 users from 40 countries (43% users from USA, 38% from EU, 19% from the remaining world)*.

IMGT color menu for the CDR-IMGT of a V-DOMAIN indicates the type of rearrangement V–J or V–D–J (2, 3). Thus, the IMGT color menu for CDR1-IMGT, CDR2-IMGT, and CDR3-IMGT is red, orange, and purple for the IG VH and for the TR V-BETA or V-DELTA (encoded by a V–D–J-REGION resulting from a V–D–J rearrangement), and blue, green, and greenblue for the IG V-KAPPA or V-LAMBDA and for the TR V-ALPHA or V-GAMMA (encoded by a V–J-REGION resulting from a V–J rearrangement). Arbitrarily the red, orange, and purple is used for the BC, C′C″ and FG loops of the V-domain of IgSF other than IG or TR.

The IMGT/Collier-de-Perles tool is integrated in IMGT/ DomainGapAlign (12, 27, 28) (users start from V, C, or G AA sequences) and in IMGT/V-QUEST (15–20) (users start from IG and TR V-DOMAIN nucleotide sequences) (Table 8). IMGT Colliers de Perles for V, C, and G-domains are provided in IMGT/2Dstructure-DB (for AA sequences in the database), and in IMGT/3Dstructure-DB (on two layers with hydrogen bonds for the V or C-domains or with the pMH contact sites for the G-domains, for 3D structures in the database) (11–13) (Table 8).

## IMGT^®^ Tools for IG, TR, and MH Domain Analysis

### IMGT/V-QUEST

#### IMGT/V-QUEST for IG and TR V-domain analysis

IMGT/V-QUEST (15–20) is the IMGT^®^ online tool for the analysis of nucleotide sequences of the IG and TR V-DOMAIN (Table 8). IMGT/V-QUEST identifies the variable (V), diversity (D), and junction (J) genes in rearranged IG and TR sequences and, for the IG, the nucleotide (nt) mutations and AA changes resulting from somatic hypermutations by comparison with the IMGT/V-QUEST reference directory. The tool integrates IMGT/JunctionAnalysis (21, 22) for the detailed characterization of the V–D–J or V–J junctions, IMGT/Automat (23, 24) for a complete sequence annotation, and IMGT/ Collier-de-Perles (29).

The IMGT/V-QUEST most important functionalities include: introduction of IMGT gaps in the user nucleotide sequences (and in its translation), alignments, and identification of the genes and alleles with the closest germline V, D, and J genes, analysis of the junctions, analysis of somatic hypermutations, and AA changes and, if the option “Search for insertions and deletions” was selected, identification of insertions and deletions (indels) and their correction. Customized parameters and results provided by IMGT/V-QUEST and IMGT/JunctionAnalysis have been described elsewhere (15–20).

#### IMGT/V-QUEST reference directory

The IMGT/V-QUEST reference directory sets against which the IMGT/V-QUEST is running include IMGT reference sequences from all functional (F) genes and alleles, all ORF and all in-frame pseudogenes (P) alleles. By definition, the IMGT reference directory sets contain one sequence for each allele. By default, the user sequences are compared with all genes and alleles. However, the option “With allele *01 only” is useful for: (i) “Detailed view,” if the user sequences need to be compared with different genes, and (ii) “Synthesis view,” if the user sequences, which use the same gene need to be aligned together (independently of the allelic polymorphism) (17, 19).

The IMGT/V-QUEST reference directories have been set up for species, which have been extensively studied, such as human and mouse. This also holds for the other species or taxons with incomplete IMGT reference directory sets. In those cases, results should be interpreted considering the status of the IMGT reference directory (information on the updates on the IMGT^®^ Web site). Links to the IMGT/V-QUEST reference directory sets are available from the IMGT/V-QUEST Welcome page (17, 19).

### IMGT/HighV-QUEST

#### NGS IG and TR V-domain analysis

IMGT/HighV-QUEST (25), created in October 2010, is the high-throughput version of IMGT/V-QUEST. It is so far the only online tool available on the Web for the direct analysis of complete IG and TR domain sequences from NGS. It analyzes sequences obtained from the Roche 454 Life Sciences technology, without the need of computational read assembly (25, 26). IMGT/HighV-QUEST analyses up to 500,000 sequences per run in November 2013 (25, 26), with the same degree of resolution and high-quality results as IMGT/V-QUEST (15–20). IMGT/HighV-QUEST represents a major breakthrough for the analysis and the comparison of the antigen receptor V-DOMAIN repertoires and immunoprofilings of the adaptive immune response (25, 26).

The functionalities of IMGT/HighV-QUEST include: the introduction of IMGT gaps, the identification of indels and their correction (19) (by default), the identification of the closest V, D, and J genes and alleles, the IMGT/JunctionAnalysis results, the description of mutations and AA changes, the annotation by IMGT/Automat, the NGS statistical analysis, and the characterization of the IMGT clonotypes (AA) (25, 26) (Table 8). IMGT/HighV-QUEST provides results in different categories “1 copy” and “More than 1” to avoid redundancy of the analysis, “single allele” and “several alleles (or genes)” (with “single allele” sequences being usually longer than “several alleles”) (25). These categories have been fundamental in the characterization of clonotypes for NGS (26).

As for the other IMGT^®^ databases and tools, IMGT/HighV-QUEST is freely available for academics. However, the IMGT/HighV-QUEST Welcome page requires user identification and provides, for new users, a link to register. User identification has been set to avoid non-relevant use and overload of the server, and to contact the user if needed. The user identification gives access to the IMGT/HighV-QUEST Search page.

#### NGS IMGT^®^ clonotype identification

##### IMGT clonotype (AA) identification: clonal diversity

In the literature, clonotypes are defined differently, depending on the experiment design (functional specificity) or available data. Thus, a clonotype may denote either a complete antigen receptor (e.g., IgG1-kappa), or only one of the two chains of the receptor (e.g., H or L), or one domain (e.g., VH), or the CDR3 sequence of a domain. Moreover the sequence can be at the AA or nucleotide (nt) level, and this is rarely specified. Therefore, IMGT^®^ goal was first of all to define clonotypes and their properties, which could be identified and characterized by IMGT/HighV-QUEST, unambiguously (26).

In IMGT^®^, the clonotype, designated as “IMGT clonotype (AA),” is defined by a unique V-(D)-J rearrangement (with IMGT gene and allele names determined by IMGT/HighV-QUEST at the nt level) and a unique CDR3-IMGT AA (in-frame) junction sequence (26). For identifying “IMGT clonotypes (AA)” in a given IMGT/HighV-QUEST dataset, the “1 copy” are filtered to select for sequences with in-frame junction, conserved anchors 104 and 118 (“C” is 2nd-CYS 104, and “F” or “W” is the J-PHE or J-TRP 118) and for V and J functional or ORF, and “single allele” (for V and J) (26).

By essence, an “IMGT clonotype (AA)” is “unique” for a given dataset. For that reason, each “IMGT clonotype (AA),” in a given dataset, has a unique set identifier (column “Exp. ID”) and, importantly, has a unique representative sequence (link in column “Sequence ID”) selected by IMGT/HighV-QUEST among the “1 copy” “single allele” (for V and J), based on the highest percent of identity of the V-REGION (“V%”) compared to that of the closest germline, and/or on the sequence length (thus the most complete V-REGION) (26).

##### Sequences assigned to IMGT clonotypes (AA): clonal expression

Clonal expression is the number of sequences that can be assigned to each IMGT clonotype (AA). In our procedure, the high-quality and specific characterization of the “IMGT clonotype (AA)” (26) remains unaltered whereas the total number of sequences assigned to each given “IMGT clonotype (AA)” is calculated stepwise by adding:

1. The number of the “1 copy” “single allele” sequences not selected as representative. These sequences differ from the representative sequence by a different (usually shorter) length, and/or by sequencing errors in the V-REGION (lower “V%” of identity) or in the J-REGION, and/or by nt differences in the CDR3-IMGT. Sequences with nt differences in the CDR3-IMGT are identified as “IMGT clonotypes (nt),” the nt differences resulting from sequencing errors or, if this can be proven experimentally, from molecular convergence. For a given “IMGT clonotype (AA),” the number (nb) of different CDR3-IMGT (nt) or “IMGT clonotypes (nt),” the CDR3-IMGT sequence (nt) and the nb of different nt in the CDR3-IMGT are reported in the results (26).
2. The number of the “1 copy” “several alleles (or genes)” sequences that have the same V and J allele as the IMGT clonotype (AA), among their IMGT/HighV-QUEST results.
3. The number of “More than 1” (including those of the IMGT representative sequence) for each retained “1 copy” of steps 1 and 2 (25).

For the first time for NGS antigen receptor data analysis, the IMGT^®^ standardized approach allows a clear distinction and accurate evaluation between the clonal diversity [nb of “IMGT clonotypes (AA)”], and the clonal expression [total nb of sequences assigned, unambiguously, to a given “IMGT clonotype (AA)”] (26). These assignments are clearly described and visualized in detail so the user always has the means of checking clonotypes individually. Indeed, the sequences of each “1 copy” assigned to a given “IMGT clonotype (AA)” are available in “Sequences file” (26). The user can easily perform an analysis of these sequences online with IMGT/V-QUEST (up to 10 sequences, selecting “Synthesis view display” and the option “Search for insertions and deletions”) and/or with IMGT/JunctionAnalysis (up to 5,000 junction sequences), which provide a visual representation familiar to the IMGT^®^ users.

Clonal diversity is also visualized in the online results with histograms, which represent the number of IMGT clonotypes (AA) per V, D (for IGH, TRB or TRD), and J genes (in pink) (26). Clonal expression is visualized with histograms, which represent the number of sequences assigned to IMGT clonotypes (AA) per V (in green), D (in red), and J (in yellow) genes (26). Values are normalized, respectively, for 10,000 IMGT clonotypes (AA) to represent IG diversity immunoprofiles per V, D (for IGH, TRB or TRD), and J genes, and for 10,000 sequences assigned to IMGT clonotypes (AA) to represent IG expression immunoprofiles per V, D (for IGH, TRB or TRD), and J genes (26). These normalized values allow comparative analysis studies performed with the same IMGT/HighV-QUEST standards (26).

### IMGT/DomainGapAlign

#### V, C, and G-domain analysis of IG, TR, and MH

IMGT/DomainGapAlign (12, 27, 28) is the IMGT^®^ online tool for the analysis of AA sequences and 2D structures of V and C-domains (for IG, TR, and other IgSF) and of G-domains (for MH and other MhSF) (Table 8). It analyzes domain AA sequences by comparison with the IMGT domain reference directory sets (translation of the germline V and J genes and of the C gene domains for IG and TR, AA domain sequences of MH and conventional genes). IMGT/DomainGapAlign functionalities include: introduction of IMGT gaps in the user AA sequences, alignments, and identification of the genes and alleles by comparison with the closest domain(s), delimitation of the domain(s) (e.g., V, C, or G) in the user sequence, description of the AA changes and IMGT Collier de Perles.

#### IMGT domain reference directory

The IMGT domain reference directory is the IMGT reference directory for V, C, and G-domains. Sequences are from the IMGT Repertoire (1) and from IMGT/GENE-DB (10). Owing to the particularities of the V-DOMAIN synthesis (2, 3), there is no V-DOMAIN in the IMGT reference directory. Instead, the directory comprises the translation of the IG and TR germline V and J genes (V-REGION and J-REGION, respectively). The IMGT domain reference directory provides the IMGT^®^ “gene” and “allele” names. Data are comprehensive for human and mouse IG and TR whereas for other species and other IgSF and MhSF they are added progressively. The IMGT domain reference directory comprises domain sequences of functional (F), ORF, and in-frame pseudogene (P) genes. As IMGT^®^ alleles are characterized at the nucleotide level, identical sequences at the AA level may therefore correspond to different alleles, in the IMGT domain reference directory. The sequences can be displayed by querying IMGT/DomainDisplay (see footnote text 1).

## IMGT^®^ Databases for IG, TR, and MH Domain Analysis

### IMGT/3Dstructure-DB

#### IMGT/3Dstructure-DB card

IMGT/3Dstructure-DB (11–13), the IMGT^®^ structure database, provides IMGT^®^ annotation and contact analysis of IG, TR, MH, IgSf, and MhSF 3D structures, and paratope/epitope description of IG/antigen and TR/pMH complexes (Table 8). There is one “IMGT/3Dstructure-DB card” per IMGT/3Dstructure-DB entry and this card provides access to all data related to that entry. The “PDB code” (four letters and/or numbers, e.g., 1n0x) is used as “IMGT entry ID” for the 3D structures obtained from the Research Collaboratory for Structural Bioinformatics (RCSB) Protein Data Bank (PDB) (100). The IMGT/3Dstructure-DB card provides eight search/display options: “Chain details,” “Contact analysis,” “Paratope and epitope,” “3D visualization Jmol or QuickPDB,” “Renumbered IMGT files,” “IMGT numbering comparison,” “References and links,” and “Printable card” (11–13).

#### IMGT chain and domain annotation

The “Chain details” section comprises information first on the chain itself, then per domain (11–13). Chain and domain annotation includes the IMGT gene and allele names (CLASSIFICATION), region and domain delimitations (DESCRIPTION) and domain AA positions according to the IMGT unique numbering (NUMEROTATION) (8, 62–66). The closest IMGT^®^ genes and alleles (found expressed in each domain of a chain) are identified with the integrated IMGT/DomainGapAlign (12, 27, 28), which aligns the AA sequences of the 3D structures with the IMGT domain reference directory.

#### Contact analysis

**Figure 6:**
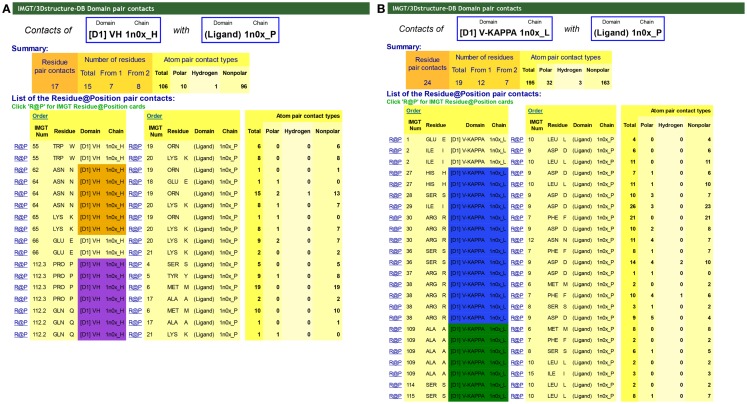
**IMGT/3Dstructure-DB Domain pair contacts between VH and V-KAPPA and the ligand from an IG/Ag complex**. The IG/Ag complex structure is 1n0x from IMGT/3Dstructure-DB (http://www.imgt.org) (11–13). The ligand is a synthetic peptide. **(A)** Domain pair contacts between VH and the ligand. The Summary shows that there are a total of 106 atom pair contacts (10 polar including 1 hydrogen bond and 96 non-polar) for 17 pair contacts between the VH (1n0x_H) and the ligand (1n0x_P). Seven amino acids (AA) of the VH interact with the ligand. The list of the pair contacts show that three of them belong to the CDR2-IMGT (orange color online) and two of them to the CDR3-IMGT (purple color online), and together contribute to 81 atom pair contacts (including the hydrogen bond). The VH binds the ligand primarily by the N64 of the CDR2-IMGT and the P112.3 and Q112.2 of the CDR3-IMGT that are localized next to the top of the loops (Figure 3). The only two positions of the FR-IMGT that have contacts with the ligand are the anchors 55 and 66 of the CDR2-IMGT. In that structure, there is no contact of the CDR1-IMGT. **(B)** Domain pair contacts between V-KAPPA and the ligand. The Summary shows that there are a total of 195 atom pair contacts (32 polar including 3 hydrogen bonds and 163 non-polar) for 24 pair contacts between the V-KAPPA (1n0x_L) and the ligand (1n0x_P). Twelve AA of the VH interact with the ligand. The list of the pair contacts show that seven of them belong to the CDR1-IMGT (blue color online) and three of them to the CDR3-IMGT (greenblue color online) and together contribute to 174 atom pair contacts (including the three hydrogen bonds). The only two positions of the FR-IMGT that have contacts with the ligand are the positions 1 and 2 of the strand A of the FR1-IMGT. In that structure, there is no contact with the CDR2-IMGT.

“Contact analysis” gives access to a table with the different “Domain pair contacts” of the 3D structure [this table is also accessed from “Chain details” by clicking on “Domain contact (overview)”]. “Domain pair contacts” refer to contacts between a pair of domains or between a domain and a ligand. Clicking on “DomPair” gives access to the contacts between AA for a given “Domain pair contacts.” Contacts between VH and the Ligand (antigen, Ag) and the V-KAPPA and the Ligand (Ag) of an IG/Ag complex are shown in Figure 6. These contact analysis representations are important as they demonstrate that most contacts with the ligand, if not all, involve the AA of the CDR-IMGT. They definitively confirmed the CDR-IMGT delimitations as the official reference standards (66, 70, 93).

In IMGT/3Dstructure-DB, all contacts are described as atom pair contacts. Atom pair contacts are obtained by a local program in which atoms are considered to be in contact when no water molecule can take place between them (11, 12). Atom pair contacts are provided by atom contact types (noncovalent, polar, hydrogen bond, nonpolar, covalent, disulfide) and/or atom contact categories [(BB) backbone/backbone, (SS) side chain/side chain, (BS) backbone/side chain, (SB) side chain/backbone] (11, 12, 90, 91).

Clicking on “R@P” gives access to the IMGT identity card of a given residue (usually an AA) at a given position or Residue@Position. The IMGT R@P card can also be accessed from the AA sequences of the IMGT/3Dstructure-DB card or from the IMGT Colliers de Perles, by clicking on one AA. In an IMGT R@P card, the Residue@Position is defined by the IMGT position numbering in a domain, or if not characterized, in the chain, the AA name (three-letter and between parentheses one-letter abbreviations), the IMGT domain description and the IMGT chain ID, e.g., “103 – TYR (Y) – VH – 1hzh_H” (11–13). The IMGT R@P card includes (i) general information (PDB file numbering, IMGT file numbering, residue full name and formula), (ii) structural information “IMGT LocalStructure@Position” [secondary structure, Phi and Psi angles (in degrees) and accessible surface area (ASA) (in square angstrom)] and (iii) detailed contact analysis with AA of other domains (11–13).

#### Paratope and epitope

In an IG/Ag complex, the AA in contact at the interface between the IG and the Ag constitute the paratope on the IG V-DOMAIN surface, and the epitope on the Ag surface. Similarly, in an TR/pMH complex, the AA in contact at the interface between the TR and the pMH constitute the paratope on the TR V-DOMAIN surface, and the epitope on the pMH surface. For IG/Ag and TR/pMH, the paratope and epitope are displayed in Contact analysis, but for each V-domain, separately. Clicking on the “Paratope and epitope” tag (displayed in the IMGT/3Dstructure-DB card, only if relevant), gives access to “IMGT paratope and epitope details”, which are described in a standardized way. Each AA that belongs to the paratope is defined by its position in a V-DOMAIN. Each AA that belongs to the epitope in an IG/Ag complex is defined by its position in the chain in the 3D structure or, if the antigen belongs to an IgSF or MhSF protein and if the epitope is part of a characterized V, C, or G-domain, by its position in the domain according to the IMGT unique numbering. The epitope in a TR/pMH complex includes AA of the peptide and of the two G-DOMAIN helices.

#### Renumbered flat file and IMGT numbering comparison

“Renumbered IMGT file” allows to view (or download) an IMGT coordinate file renumbered according to the IMGT unique numbering, and with added IMGT specific information on chains and domains (added in the “REMARK 410” lines (blue online), and identical to the “Chain details” annotation).

“IMGT numbering comparison” provides, per domain, the IMGT DOMAIN numbering by comparison with the PDB numbering, and the residue (three-letter and one-letter names), which allows standardized IMGT representations using generic tools (Figures 3A and 4A).

#### IMGT/3Dstructure-DB associated tools

Tools associated to IMGT/3Dstructure-DB include IMGT/ StructuralQuery (11) and IMGT/DomainSuperimpose, available online. IMGT/StructuralQuery allows to retrieve the IMGT/3Dstructure-DB entries, based on specific structural characteristics of the intramolecular interactions: phi and psi angles, ASA, type of atom contacts, distance in angstrom between AA, IMGT Residue@Position contacts and, for V-DOMAIN, CDR-IMGT length or pattern (11). IMGT/DomainSuperimpose allows to superimpose the 3D structures of two domains from IMGT/3Dstructure-DB.

### IMGT/2Dstructure-DB

IMGT/2Dstructure-DB was created as an extension of IMGT/3Dstructure-DB (11–13) to describe and analyze AA sequences of chains and domains for which no 3D structures were available (Table 8). IMGT/2Dstructure-DB uses the IMGT/3Dstructure-DB informatics frame and interface, which allow one to analyze, manage, and query IG, TR, and MH, as well as other IgSF and MhSF and engineered proteins (FPIA, CPCA), as polymeric receptors made of several chains, in contrast to the IMGT/LIGM-DB sequence database that analyzes and manages sequences individually (9). The AA sequences are analyzed with the IMGT^®^ criteria of standardized identification (59), description (60), nomenclature (61), and numerotation (8, 62–66).

The current IMGT/2Dstructure-DB entries include AA sequences of antibodies from Kabat (94) (those for which there were no available nucleotide sequences), and AA sequences of mAb and FPIA from the WHO–INN Programme (14, 50, 51). Queries can be made on an individual entry, using the Entry ID or the Molecule name. The same query interface is used for IMGT/2Dstructure-DB and IMGT/3Dstructure-DB. Thus a “trastuzumab” query in “Molecule name” allows to retrieve three results: two INN (“trastuzumab” and “trastuzumab emtansine”) from IMGT/2Dstructure-DB, and one 3D structure (“1nz8”) from IMGT/3Dstructure-DB.

The IMGT/2Dstructure-DB cards provide standardized IMGT information on chains and domains and IMGT Colliers de Perles on one or two layers, identical to that provided for the sequence analysis in IMGT/3Dstructure-DB, however the information on experimental structural data (hydrogen bonds in IMGT Collier de Perles on two layers, Contact analysis) is only available in the corresponding IMGT/3Dstructure-DB cards, if the antibodies have been crystallized.

## IMGT^®^ V and C-Domain for Antibody Humanization and Engineering

### CDR-IMGT delimitation for grafting

The objective of antibody humanization is to graft at the DNA level the CDR of an antibody V-domain, from mouse (or other species) and of a given specificity, onto a human V-domain framework, thus preserving the specificity of the original (murine or other species) antibody while decreasing its immunogenicity (101). IMGT/DomainGapAlign (12, 27, 28) is the reference tool for antibody humanization design based on CDR grafting. Indeed, it precisely defines the CDR-IMGT to be grafted and helps selecting the most appropriate human FR-IMGT by providing the alignment of the AA sequences between the mouse (or other species) and the closest human V-DOMAIN.

Analyses performed on humanized therapeutic antibodies underline the importance of a correct delimitation of the CDR and FR. As an example, two AA changes were required in the first version of the humanized VH of alemtuzumab, in order to restore the specificity and affinity of the original rat antibody. The positions of these AA changes (S28 > F and S35 > F) are now known to be located in the CDR1-IMGT and should have been directly grafted, but at the time of this mAb humanization they were considered as belonging to the FR according to the Kabat numbering (94). In contrast, positions 66–74 were, at the same time, considered as belonging to the CDR according to the Kabat numbering, whereas they clearly belong to the FR2-IMGT and the corresponding sequence should have been “human” instead of being grafted from the “rat” sequence (IMGT^®^, see footnote text 1, The IMGT Biotechnology page > Antibody humanization > Alemtuzumab).

### IGHG1 alleles and G1m allotypes

Allotypes are polymorphic markers of an IG subclass that correspond to AA changes and are detected serologically by antibody reagents (76). In therapeutic antibodies (human, humanized, or chimeric) (14), allotypes may represent potential immunogenic residues (75), as demonstrated by the presence of antibodies in individuals immunized against these allotypes (76). The allotypes of the human heavy gamma chains of the IgG are designated as Gm (for gamma marker).

The allotypes G1m, G2m, and G3m are carried by the constant region of the gamma1, gamma2, and gamma3 chains, encoded by the IGHG1, IGHG2, and IGHG3 genes, respectively (76). The gamma1 chains may express four G1m alleles (combinations of G1m allotypes): G1m3, G1m3,1, G1m17,1, and G1m17,1,2 (and in Negroid populations two additional G1m alleles, Gm17,1,28 and Gm17,1,27,28) (76) (Table 9). The C-region of the G1m3,1, G1m17,1, and G1m17,1,2 chains differ from that of the G1m3 chains by two, three, and four AA, respectively (76). The correspondence between the G1m alleles and IGHG1 alleles is shown in Table 9. Thus, IGHG1*01 and IGHG1*02 are G1m17,1, IGHG1*03 is G1m3, IGHG1*04 is G1m17,1,2 and IGHG1*05 is G1m3,1.

**Table 9:** **Correspondence between the IGHG1 alleles and G1m alleles**.

IGHG1 alleles	G1m alleles^a^		IMGT amino acid (AA) positions^b^					Populations (76)
	Allotypes	Isoallotypes^c^	CH1		CH3		
			**103**	120	12	14	110	
							
			G1m3^d^	G1m17/nG1m1	G1m1/nG1m1		G1m2/-
IGHG1*01^e^	G1m17,1		I	**K**	**D**	**L**	A	Caucasoid Negroid
IGHG1*02^e^								Mongoloid
IGHG1*03	G1m3	*nG1m1*, *nG1m17*	**I**	**R**	E	M	A	Caucasoid
IGHG1*04^f^	G1m17,1,2		I	**K**	**D**	**L**	**G**	Caucasoid
								Mongoloid
IGHG1*05^f^	G1m3,1	*nG1m17*	**I**	**R**	**D**	**L**	A	Mongoloid

*^a^In Negroid populations, the G1m17,1 allele frequently includes G1m27 and G1m28, leading to two additional G1m alleles, G1m17,1,27 and G1m17,1,27,28 (76)*.

*^b^AA corresponding to G1m allotypes are shown in bold*.

*^c^The nG1m1 and nG1m17 isoallotypes present on the Gm1-negative and Gm17-negative gamma1 chains (and on other gamma chains) are shown in italics*.

*^d^The presence of R120 is detected by anti-nG1m17 antibodies whereas the simultaneous presence of I103 and R120 in the gamma1 chains is detected by anti-Gm3 antibodies (76)*.

^e^The IGHG1*01 and IGHG1*02 alleles only differ at the nucleotide level (codon 85.1 in CH2).

^f^IGHG1*04 and IGHG1*05 AA are expected (76) but not yet sequenced at the nucleotide level and therefore the IGHG1*04 and IGHG1*05 alleles are not shown in IMGT Repertoire, Alignments of alleles: *Homo sapiens* IGHG1 (http://www.imgt.org).

In the IGHG1 CH1, the lysine at position 120 (K120) in strand G corresponds to the G1m17 allotype (76) (Figure 4D). The isoleucine I103 (strand F) is specific of the gamma1 chain isotype. If an arginine is expressed at position 120 (R120), the simultaneous presence of R120 and I103 corresponds to the expression of the G1m3 allotype (76). For the gamma3 and gamma4 isotypes (which also have R120 but T in 103), R120 only corresponds to the expression of the nG1m17 isoallotype (an isoallotype or nGm is detected by antibody reagents that identify this marker as an allotype in one IgG subclass and as an isotype for other subclasses) (76).

In the IGHG1 CH3, the aspartate D12 and leucine L14 (strand A) correspond to G1m1, whereas glutamate E12 and methionine M14 correspond to the nG1m1 isoallotype (76) (Table 9). A glycine at position 110 corresponds to G1m2, whereas an alanine does not correspond to any allotype (G1m2-negative chain) (Table 9). Therapeutic antibodies are most frequently of the IgG1 isotype, and to avoid a potential immunogenicity, the constant region of the gamma1 chains are often engineered to replace the G1m3 allotype by the less immunogenic G1m17 (CH1 R120 > K) (G1m17 is more extensively found in different populations) (76).

## Conclusion

IMGT-ONTOLOGY and the IMGT^®^ information system, which are at the origin of immunoinformatics, have provided the concepts, the knowledge environment, and the informatics frame for a standardized and integrated analysis of IG, TR, and MH, extended to other IgSF (102–106) and MhSF (96, 97), from gene to structure and function (32–47). IG and TR repertoire analysis, antibody humanization, IG and TR engineering for immunotherapy, paratope/epitope characterization represent major current fields of immunoinformatics at the forefront of basic, clinical, and pharmaceutical research owing to major methodological advances and medical implications.

The IMGT^®^ standards are used in clinical applications. Thus, IMGT/V-QUEST is frequently used by clinicians for the analysis of IG somatic hypermutations in leukemia, lymphoma, and myeloma, and more particularly in chronic lymphocytic leukemia (CLL) (18, 72–74) in which the percentage of mutations of the rearranged IGHV gene in the VH of the leukemic clone has a prognostic value for the patients. For this evaluation, IMGT/V-QUEST is the standard recommended by the European Research Initiative on CLL (ERIC) for comparative analysis between laboratories (72). The sequences of the V–(D)–J junctions determined by IMGT/JunctionAnalysis (21, 22) are also used in the characterization of stereotypic patterns in CLL (73, 74) and for the synthesis of probes specific of the junction for the detection and follow-up of minimal residual diseases (MRD) in leukemias and lymphomas. A new era is opening in hemato-oncology with the use of NGS for analysis of the clonality and MRD identification, making IMGT^®^ standards use more needed as ever. More generally, the IMGT/HighV-QUEST web portal is a paradigm for identification of IMGT clonotype diversity and expression in NGS immune repertoire analysis of the adaptive immune response in infectious diseases, in vaccination, and for next-generation repertoire immunoprofiling (26).

The therapeutic monoclonal antibody engineering field represents the most promising potential in medicine. A standardized analysis of IG genomic and expressed sequences, structures, and interactions is crucial for a better molecular understanding and comparison of the mAb specificity, affinity, half-life, Fc effector properties, and potential immunogenicity. IMGT-ONTOLOGY concepts have become a necessity for IG loci description of newly sequenced genomes, antibody structure/function characterization, antibody engineering [single chain Fragment variable (scFv), phage displays, combinatorial libraries] and antibody humanization (chimeric, humanized, and human antibodies) (35, 42, 44, 46, 75–77, 82). IMGT^®^ standardization allows repertoire analysis and antibody humanization studies to move to novel high-throughput methodologies with the same high-quality criteria. The CDR-IMGT lengths are now required for mAb INN applications and are included in the WHO–INN definitions (51), bringing a new level of standardized information in the comparative analysis of therapeutic antibodies.

## Availability and Citation

Authors who use IMGT^®^ databases and tools are encouraged to cite this article and to quote the IMGT^®^ Home page, http://www.imgt.org. Online access to IMGT^®^ databases and tools are freely available for academics and under licenses and contracts for companies.

## Conflict of Interest Statement

The author declares that the research was conducted in the absence of any commercial or financial relationships that could be construed as a potential conflict of interest.

## Footnotes

^1^http://www.imgt.org

^2^http://bioportal.bioontology.org/ontologies/1491
